# Supplementary material for: The Phenylpropanoid Gatekeeper CtPAL1 Coordinates ABA-Induced Flavonoid Biosynthesis and Oxidative Stress Tolerance in Safflower (Carthamus tinctorius L.)
Source: Plants (Basel). 2025 Nov 26;14(23):3606. doi: 10.3390/plants14233606 (PMC12694366; doi:10.3390/plants14233606)
Supplement: Supplementary file 1 [file plants-14-03606-s001.zip › plants-3958595-supplementary.pdf]

Supplementary Table S1. Physicochemical properties of CtPAL1.

**CDS length（bp）**	**2124**
**Molecular Weight（KD）**	**76.8**
**Isoelectric Point**	**5.84**
**Instability Index**	**34.99**
**Aliphatic index**	**91.77**
**Grand average of hydropathicity（GRAVY）**	**-0.153**

Supplementary Table S2. Amino acid profile and proportion distribution of CtPAL1-encoding protein.

Amino acid residue	Number	Proportion（%）
Ala(A)	65	9.2
Arg(R)	30	4.2
Asn(N)	33	4.7
Asp(D)	32	4.5
Cys(C)	8	1.1
Gln(Q)	25	3.5
Glu(E)	51	7.2
Gly(G)	59	8.3
His(H)	20	2.8
Ile(I)	37	5.2
Leu(L)	77	10.9
Lys(K)	39	5.5
Met(M)	18	2.5
Phe(F)	24	3.4
Pro(P)	28	4.0
Ser(S）	51	7.2
Thr(T)	42	5.9
Trp(W)	4	0.6
Tyr(Y)	16	2.3
Val(V)	48	6.8
